# Application of robotic-assisted hepatopancreatoduodenectomy in non-jaundiced gallbladder cancer patient with invasion of the common bile duct: A case report

**DOI:** 10.1097/MD.0000000000049902

**Published:** 2026-07-31

**Authors:** Kai Xu, Guodong Chen, Chen Liu, Wanghong Li, Xin Li, Guilin Cao, Longhua Zhang, Jintian Tang, Chao Yi, Feng Xue, Liping Liang, Deliang Cao, Boqing Wang

**Affiliations:** a Department of Hepatopancreatobiliary Surgery, The Affiliated Tumor Hospital of Xinjiang Medical University, Urumqi, Xinjiang Uygur Autonomous Region, China; b The Third Clinical Medical College of Xinjiang Medical University, Urumqi, Xinjiang Uygur Autonomous Region, China; c Department of Hepatopancreatobiliary Surgery, The First Affiliated Hospital & Key Laboratory of Intelligent Diagnosis and Treatment of Hepatobiliary and Pancreatic Tumors in Hunan Provincial Universities & Hunan Engineering Research Center for Early Diagnosis and Treatment of Liver Cancer, Hengyang Medical School, University of South China, Hengyang, Hunan, China; d Department of Pathology, Affiliated Tumor Hospital of Xinjiang Medical University, Urumqi, Xinjiang Uygur Autonomous Region, China; e Hunan Engineering Research Center for Early Diagnosis and Treatment of Liver Cancer, Hunan Province Key Laboratory of Tumor Cellular and Molecular Pathology, Cancer Research Institute, Hengyang Medical School, University of South China, Hengyang, Hunan, China.

**Keywords:** CBD invasion and case report, da Vinci Xi system, hepatopancreatoduodenectomy, minimally invasive surgery, non-jaundiced gallbladder cancer, robot-assisted HPD

## Abstract

**Rationale:**

Hepatopancreatoduodenectomy (HPD) is a surgical procedure utilized for the curative-intent resection of biliary tract malignancies. However, there is a paucity of literature documenting the application of robotic-assisted HPD in case of gallbladder cancer (GBC) with invasion into the common bile duct (CBD) in patients who had no jaundice.

**Patient concerns:**

One week before admission, a 58-year-old female suffered from paroxysmal diarrhea and vomiting without any apparent cause.

**Diagnoses:**

Preoperative computed tomography (CT) and magnetic resonance imaging revealed GBC with dilation of the CBD and common hepatic duct. Intraoperative choledochoscopy revealed a soft tissue mass (0.5–1 cm) at the distal CBD near the duodenal papilla with circumferential involvement. Given the primary GBC, metastatic involvement of the distal CBD was suspected.

**Interventions:**

After discussion, robotic-assisted HPD by the da Vinci Xi system was performed. The surgery lasted 580 minutes with 200 mL of blood loss. Postoperative pathology indicated malignant tumors of the gallbladder and bile duct.

**Outcomes:**

The patient’s postoperative vital signs were stable. The CT scan results from the one-year postoperative follow-up showed no signs of tumor recurrence or metastasis.

**Lessons:**

This case supports the viability and safety of the robotic approach to HPD for complex biliary malignancies, establishing it as a feasible minimally invasive strategy in well-selected cases, contingent upon surgical expertise.

## 1. Introduction

Hepatopancreatoduodenectomy (HPD), a procedure initially described in 1974 for the treatment of locally advanced gallbladder cancer (GBC), remains confined to highly specialized surgical centers due to its technical complexity, significant morbidity, and considerable mortality.^[1]^ Refinements in surgical anatomy, technique, and perioperative care have rendered HPD a feasible option for selected cholangiocarcinomas involving the biliary tract from the hepatic hilum to the intrapancreatic common bile duct (CBD).^[2,3]^ These advancements have not only improved oncological outcomes but also markedly reduced postoperative morbidity and mortality. Of note, complication rates declined to 31%.^[4]^

Robotic surgery is being employed across various surgical disciplines due to its benefits in visualization, dexterity, and ergonomics.^[5]^ Using the Da Vinci system, precise vascular control and suturing were achieved within a confined operative field.^[6]^ Furthermore, its features, including magnified stable three-dimensional views, vibration-filtered robotic arms, wristed instruments, and precise dissection capabilities, enable robotic surgery to achieve a balance between minimal trauma and radical resection.^[7]^ Although minimally invasive surgical methods have significantly improved the complex HPD, the application of da Vinci robot-assisted HPD has rarely been reported.^[8]^

To the best of our knowledge, this study is the first to report the treatment of cases involving GBC and CBD invasion using the Da Vinci Xi system. This case aims to demonstrate the technical feasibility and safety of HPD assisted by robot system, which helps to accumulate more evidence in support of its application in complex liver, gallbladder, and pancreas surgeries.

## 2. Case presentation

**Figure 1.**
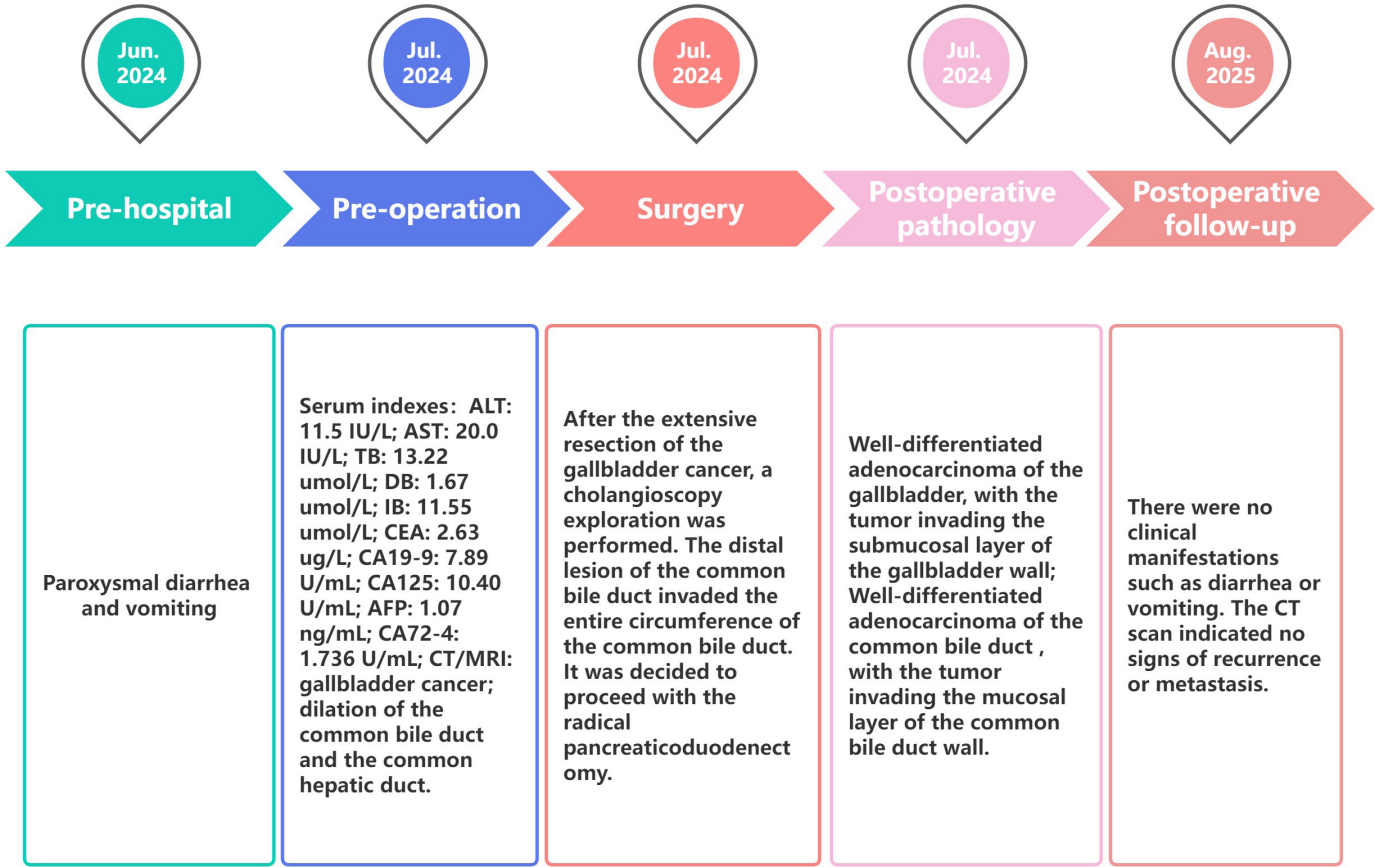
Timeline of clinical visits in this case. The infographic illustrates the chronological sequence of clinical visits and diagnoses. AFP = alpha-fetoprotein, ALT = alanine aminotransferase, AST = aspartate aminotransferase, CA19-9 = carbohydrate antigen 19-9, CA72-4 = carbohydrate antigen 72-4, CA125 = carbohydrate antigen 125, CEA = carcinoembryonic antigen, CT = computed tomography, DB = direct bilirubin, IB = indirect bilirubin, MRI = magnetic resonance imaging, TB = total bilirubin.

Ethical approval for this study was obtained from the Institutional Review Board of the Affiliated Tumor Hospital at Xinjiang Medical University (Approval No. S-2024080). Meanwhile, the patient in this study agreed to have her clinical data used for publication, and she was informed of the purpose of the study and signed the informed consent form. Figure 1 illustrated the timeline of clinical visits and the diagnostic history of this patient. A 58-year-old female presented in July 2024 with paroxysmal diarrhea and vomiting persisting for one week. Her surgical history includes appendectomy (1984), acoustic neuroma resection (2004), and intracranial cyst excision (2015). The patient had no documented family history of gastrointestinal cancers or hereditary cancer syndromes. Physical examination revealed stable vital signs and unremarkable assessments of the abdomen, cardiopulmonary system, and lymphatic system. Laboratory studies demonstrated normal hepatic function and tumor markers within reference ranges (Table 1).

**Table 1:** Preoperative serological related indicators.

Indicators	Results	Reference range
Total bilirubin	13.22	0–21 µmol/L
Direct bilirubin	1.67	0–6 µmol/L
Indirect bilirubin	11.55	0–18 µmol/L
CEA	2.63	0–5 µg/L
CA19-9	7.89	0–39 µg/L
CA125	10.40	0–35 µg/L
AFP	1.07	0–13.4 ng/mL
CA72-4	1.736	0–6.9 ng/mL

AFP = alpha-fetoprotein, CA125 = carbohydrate antigen 125, CA19-9 = carbohydrate antigen 19-9, CA72-4 = carbohydrate antigen 72-4, CEA = carcinoembryonic antigen.

**Figure 2.**
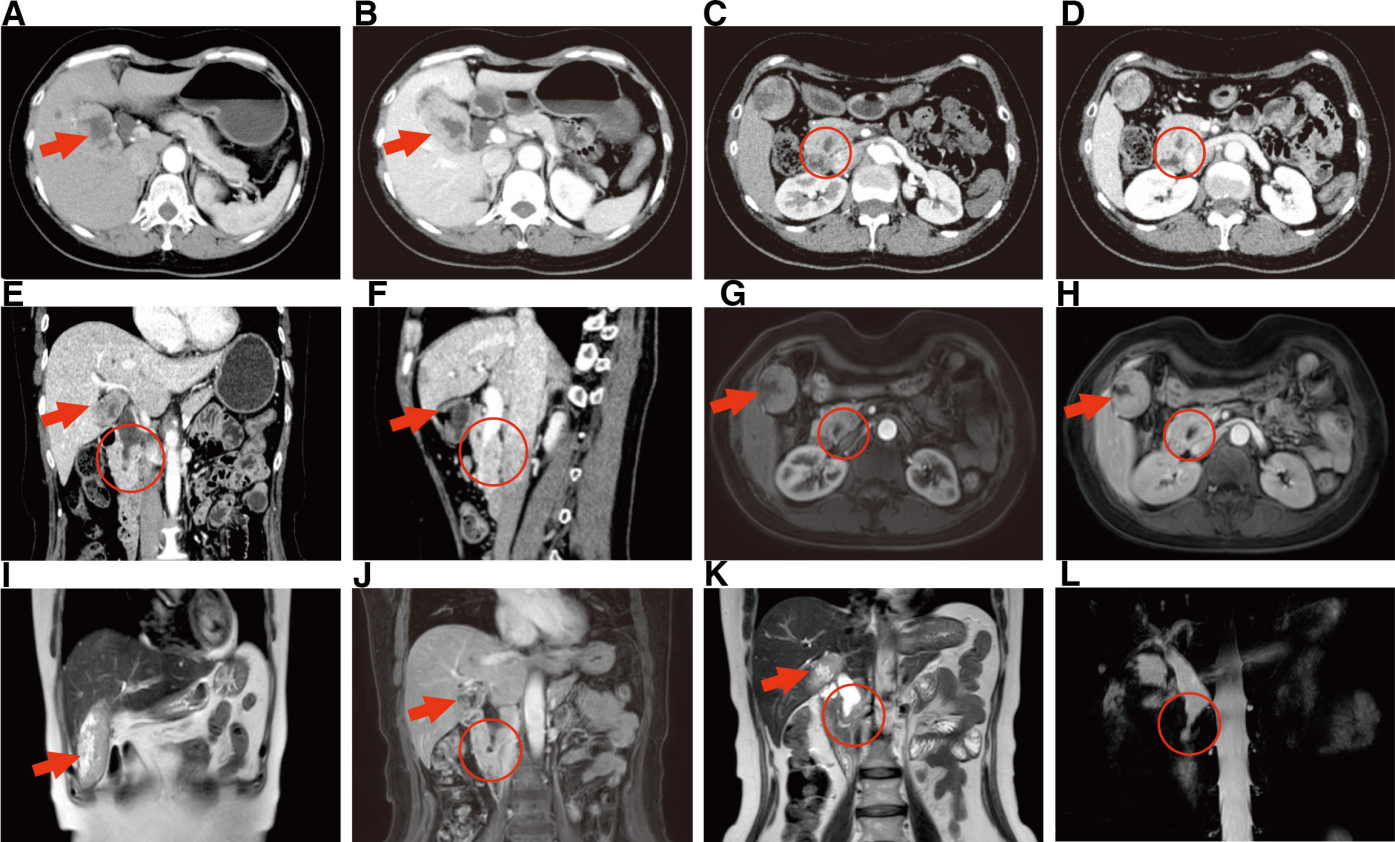
The results of the patient’s CT and MRCP. (A,B) Preoperative CT scans in the arterial (A) and venous phases (B) revealed irregular thickening of the gallbladder wall and the formation of a soft tissue mass protruding into the gallbladder cavity. The cavity was filled with irregular masses, and the boundaries were unclear. (C,D) Preoperative CT scans in the arterial (C) and venous phases (D) revealed a mass at the distal end of the common bile duct, with dilation of the proximal part. (E,F) The coronal (E) and sagittal (F) views in the CT scan indicated a space-occupying lesion within the gallbladder cavity, as well as stenosis of the distal common bile duct and dilation of the proximal common bile duct. (G,H) The MRI arterial (G) and venous phases (H) indicated an enlarged gallbladder volume. Irregular nodular thickening was observed on the base and body wall of the gallbladder, with soft tissue masses protruding into the gallbladder cavity. After enhanced scanning, the masses showed heterogeneous enhancement. (I) The MRI coronal view shows a lesion within the gallbladder. (J–L) The MRI coronal scan (J,K) and MRCP (L) indicate that the distal end of the common bile duct is narrowed, while the proximal part is dilated. The arrow indicates the lesion of the gallbladder. The circular mark indicates the lesion of the common bile duct. CT = computed tomography, MRCP = magnetic resonance cholangiopancreatography, MRI = magnetic resonance imaging.

Based on the contrast-enhanced computed tomography (CT) findings, the gallbladder wall demonstrated irregular thickening with heterogeneous enhancement during the arterial phase (Fig. 2A), followed by progressive and persistent enhancement in the venous phase (Fig. 2B). Concurrently, focal thickening at the distal CBD was observed in the arterial phase (Fig. 2C), with gradually increasing enhancement intensity in the venous phase (Fig. 2D). Further reconstructions in the coronal plane (Fig. 2E) and sagittal plane (Fig. 2F) revealed distal CBD stenosis and proximal dilatation. These features collectively indicate gallbladder wall thickening with an intraluminal soft-tissue mass, suggesting GBC with concomitant invasion of the CBD. Subsequent magnetic resonance imaging (Fig. 2G–K) and magnetic resonance cholangiopancreatography (Fig. 2L) further corroborated these diagnoses.

After a multidisciplinary discussion, surgery was performed on July 27, 2024. According to the relevant operation guidelines, anesthesia, disinfection, connection of the robotic arm, and establishment of pneumoperitoneum were carried out in sequence. After a systematic abdominal exploration, the results were as follows: The gallbladder was significantly enlarged; the CBD was dilated, and no obvious metastatic lesions were found in the abdominal cavity.

**Figure 3.**
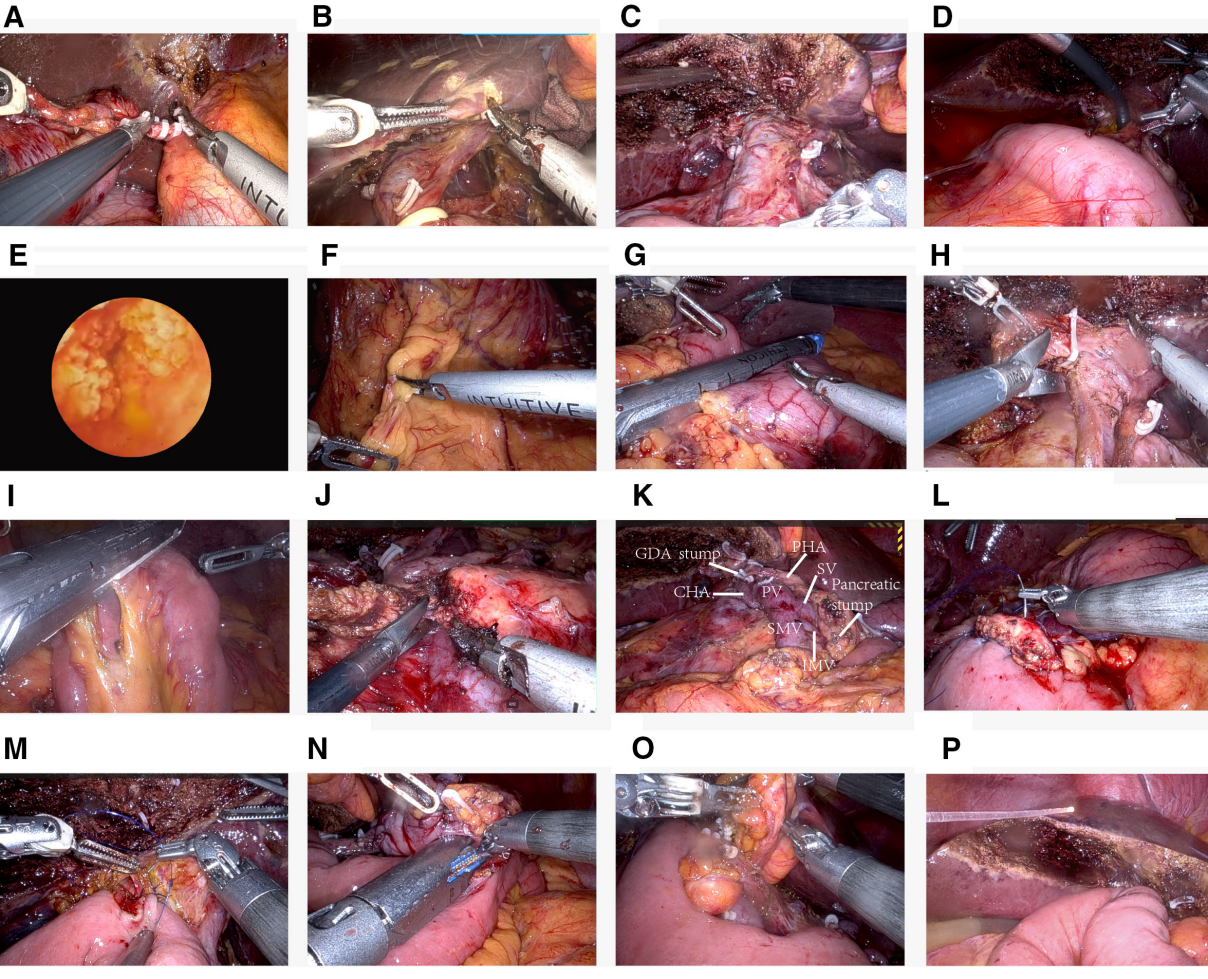
Robotic pancreatoduodenectomy procedure. (A) The cystic duct was transected. (B) The liver parenchyma located 2–3 cm from the gallbladder fossa was demarcated. (C) A wedge resection of the hepatic parenchyma in segments IVb and V was performed. (D) Choledochoscopic exploration of the biliary tree was performed. (E) An obstructing mass was identified in the distal common bile duct. (F) The gastrocolic ligament was divided. (G–J) The stomach (G), ductus hepaticus communis (H), jejunum (I), and pancreas (J) were sequentially transected. (K) The overall surgical field view after specimen transection. (L–N) Pancreaticojejunostomy (L), hepaticojejunostomy (M), and gastrojejunostomy (N) were performed sequentially. (O) Shell-like encasement of the hepatic artery. (P) Drainage pipes were placed in the surgical field. CHA = common hepatic artery, GDA stump = gastroduodenal artery stump, IMV = inferior mesenteric vein, PHA = proper hepatic artery, PV = portal vein, SV = splenic vein.

Following adhesiolysis, Calot triangle was meticulously dissected to isolate the cystic structures. The cystic artery was clipped and divided. The cystic duct was triple-clipped and transected 5 mm from the CBD (Fig. 3A). The gallbladder was excised from the hepatic bed, revealing a lumen filled with fish-fleshy nodules highly suspicious for malignancy intraoperatively, prompting frozen section analysis. Rapid pathology consultation reported moderately differentiated adenocarcinoma of the gallbladder, with cystic duct margins negative for carcinoma infiltration. Subsequently, an extended radical cholecystectomy was performed. A Pringle maneuver with intermittent hepatic vascular exclusion (15 minutes clamping and 5 minutes release) was applied. After marking liver segments IVb and V 2 to 3 cm away from the gallbladder bed (Fig. 3B), a wedge-shaped resection of liver tissue and a portal lymphadenectomy from the hepatic hilum to the pancreaticoduodenal region were performed (Fig. 3C). Concurrent intraoperative choledochoscopy was performed (Fig. 3D). Specifically, a choledochoscope was inserted via an anterior wall incision of the CBD for biliary tree exploration. Direct visualization revealed diffuse dilation of the extrahepatic bile ducts and a papillary-like mass at the lower end of the CBD (Fig. 3E). The duodenal papilla exhibited inflammatory edema, with no space-occupying lesions or impacted stones identified.

Subsequently, the gastrocolic ligament was opened (Fig. 3F), and the junction of the pancreatic neck and body was dissected. Along the celiac trunk and its bifurcation (common hepatic artery and splenic artery), lymphadenectomy of groups 7, 8, 9, and 11 was performed. Then the Helne vein was severed. Intraoperative reevaluation of the biliary system was performed. A fiberoptic choledochoscope was inserted through a longitudinal incision in the anterior wall of the CBD. Smooth mucosa and unobstructed orifices were seen in the left and right hepatic ducts and their second-order branches. A grayish-white, friable mass (1.0–1.5 cm in diameter) was found growing circumferentially in the distal CBD near the ampulla of Vater, causing luminal obstruction. Given the primary GBC, metastatic involvement of the distal CBD was suspected.

After multidisciplinary consultation and thorough communication with the family, radical pancreaticoduodenectomy was performed as follows: Following ligation of the right gastric and gastroepiploic vessels, the stomach was transected 5 cm from the pylorus on the lesser curvature with a linear stapler, and the stump was oversewn (Fig. 3G). The common hepatic duct was then ligated with hemostatic clips and transected with scissors (Fig. 3H). The jejunum was divided 10 cm distal to the ligament of Treitz using a linear stapler (Fig. 3I). Its mesentery was dissected and ligated, and the proximal jejunum was passed behind the superior mesenteric artery (SMA) to the right upper quadrant. The pancreatic uncinate process was dissected along the superior mesenteric vein (SMV); its tributaries to the SMA and SMV were ligated and divided, followed by transection of the pancreatic neck (Fig. 3J). An extended retroperitoneal lymphadenectomy was performed from the anterior inferior vena cava to the left border of the aorta. The hepatoduodenal ligament was dissected, the gastroduodenal artery was doubly ligated and divided, and the CBD was transected 1 cm below the confluence and marked. The proximal jejunum was mobilized to the right border of the SMV and brought retrocolically into the subhepatic space. Finally, the uncinate process was resected by sharp dissection along the right aspect of the SMV–portal vein axis, ligating its arterial branches from the SMA, which allowed en bloc removal of the specimen (Fig. 3K).

Digestive tract reconstruction was performed as follows: First, a distal jejunal loop was passed retrocolically to the right upper quadrant, and a properly sized stent was inserted into the pancreatic duct. After confirming the position of the pancreatic duct, a mucosa-to-mucosa pancreatojejunostomy was performed between the pancreatic remnant and the jejunal side wall (Fig. 3L). Second, 10 cm distal to the pancreatojejunostomy, a choledochojejunostomy was performed with continuous suturing between the ductuli hepaticus communis and the jejunal side wall (Fig. 3M). Third, 60 cm distal to the choledochojejunostomy, a gastrojejunostomy was performed using a disposable endoscopic linear stapler on the posterior wall of the stomach (Fig. 3N). All anastomoses and closure sites were reinforced and buried, thereby completing the gastrointestinal reconstruction. Finally, we wrapped the hepatic artery and the stump of the gastroduodenal artery (GDA) in a shell-like manner for hemostasis, protection, and prevention of postoperative bleeding (Fig. 3O).

Following irrigation of the abdominal cavity and meticulous confirmation of hemostasis at the surgical site, with no active bleeding or pancreatic leakage observed, hemostatic agents were applied to the pancreatic remnant and the operative field. One drainage pipe was placed below the pancreas, one above the choledochojejunostomy, and one below it (Fig. 3P). All were exteriorized through abdominal wall puncture sites. After ensuring no items were left inside by checking sponges and instruments, the abdomen was closed in layers, and the surgery was concluded. The operation time for the robotic surgery was 580 minutes. There was a small amount of bleeding during the procedure, but no blood transfusion was required.

**Figure 4.**
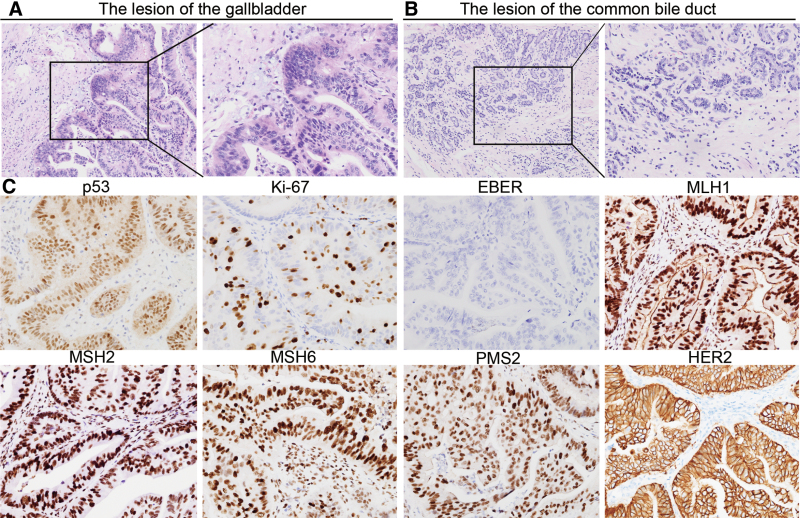
Histopathological analysis of the resected lesions. (A,B) HE staining was used to evaluate the gallbladder mass (A) and the common bile duct lesion (B). (C) Immunohistochemistry was implemented to evaluate the markers in gallbladder cancer, including p53, MLH1, MSH2, MSH6, PMS2, Ki-67, Her-2, and EBER. EBER = Epstein–Barr virus-encoded RNA.

**Figure 5.**
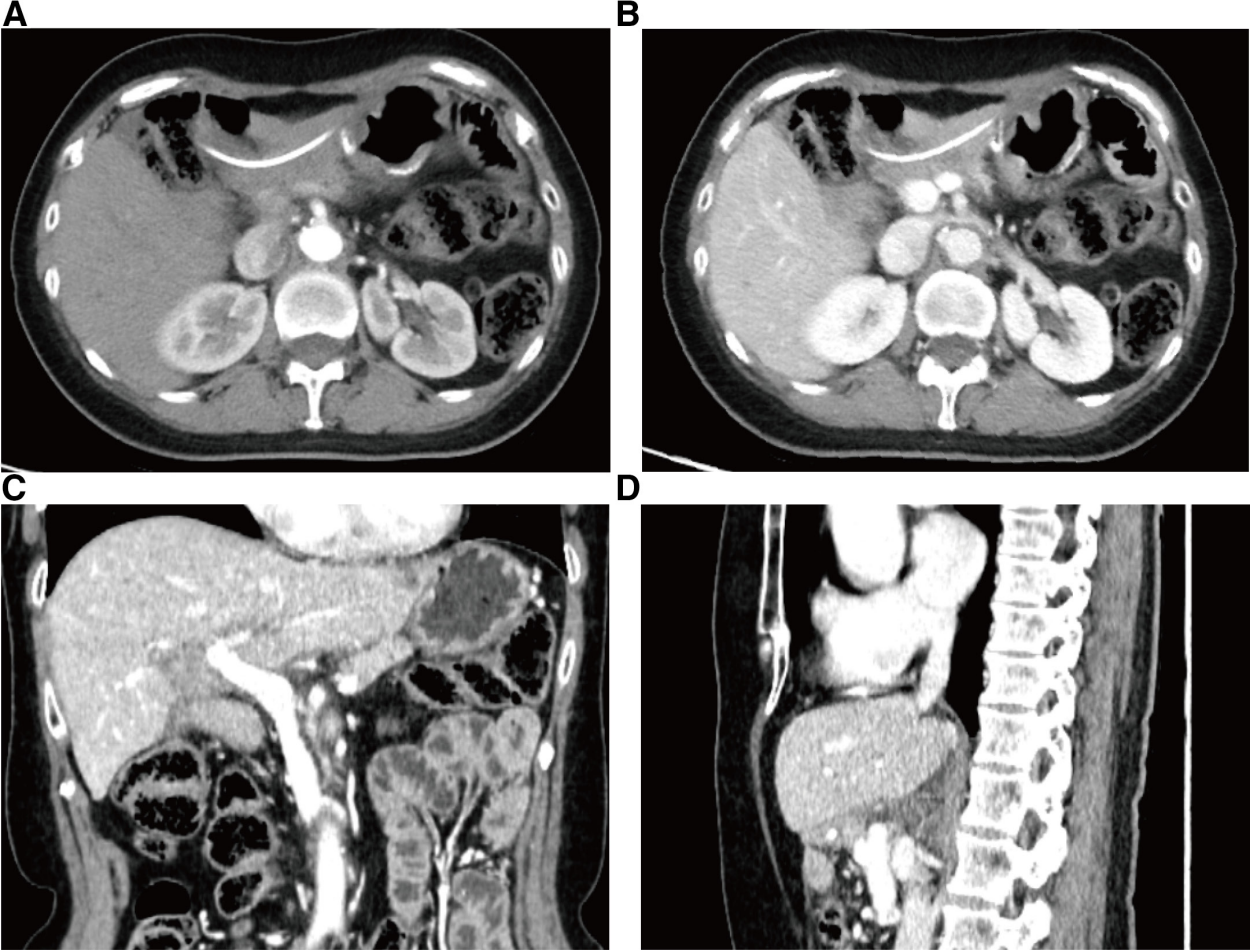
The CT scan image of the patient one year after the operation. (A,B) The CT scan images of the enhanced arterial (A) and venous phases (B) of the patient’s surgical area. (C,D) The CT scan images of the patient’s surgical area in the sagittal (C) and coronal positions (D). CT = computed tomography.

The histopathological assessment revealed that the morphology of the gallbladder lesion (Fig. 4A) and the CBD lesion (Fig. 4B) was similar, as indicated by the hematoxylin-eosin staining results. Postoperative pathology confirmed moderately differentiated adenocarcinoma of the gallbladder, staged as pT2aN0M0. Immunohistochemical profiling demonstrated (Fig. 4C): Aggressive molecular phenotype: p53-mutant pattern (diffuse strong nuclear staining in >80% tumor cells); Intact DNA repair machinery: Retained expression of all mismatch repair proteins (MLH1+, MSH2+, MSH6+, PMS2+); High proliferative activity: Ki-67 labeling index of 40% in tumor hotspots; Actionable therapeutic target: HER2 overexpression (3+ with complete intense membranous staining); Viral etiology exclusion: Epstein–Barr virus-encoded RNA in situ hybridization negative. These findings collectively define a p53-mutated/MSS (microsatellite stable)/HER2-positive molecular subtype, warranting consideration for anti-HER2 targeted therapy in adjuvant settings while indicating limited benefit from PD-1/PD-L1 inhibitors. Margin clearance >1 mm and absence of perineural invasion on ductal margins support R0 resection completeness. This patient underwent a one-year follow-up. The CT scan results indicated that no recurrence or metastasis of the tumor was observed during the arterial and venous phases (Fig. 5A and B). No bile duct stenosis or dilation was observed in the coronal and sagittal views (Fig. 5C and D). In conclusion, this case demonstrates the applicability and safety of the Da Vinci Xi system in performing HPD surgeries.

## 3. Discussion

Intracholecystic papillary neoplasm is predominantly observed in elderly female patients.^[9]^ Macroscopically, the tumor typically presents as polypoid or papillary lesions projecting into the gallbladder lumen, with either pedunculated or sessile bases.^[10,11]^ These lesions often exhibit well-defined margins and may demonstrate calcification or necrotic changes. Histologically, the tumor cells form distinct papillary or villous architectures supported by fibrovascular cores.^[12]^ Compared with other subtypes of GBC, papillary GBC predominantly exhibits exophytic growth patterns confined to the lumen, with relatively limited invasiveness into the gallbladder wall.^[13]^ In this study, the inner wall of the gallbladder was filled with fine papillary-like substances. The surface was papillary-like, the cross-section was grayish-white, and the texture was medium. Pathological examination confirmed that this was a moderately differentiated adenocarcinoma. The tumor size extended into the submucosal layer of the gallbladder wall. No clear intravascular cancerous thrombus or nerve invasion was observed. No cancerous tissue was found at the incision margin of the cystic duct.

In addition, when facing GBC with a biliary tract mass, it is essential to differentiate it from double primary tumors.^[14]^ Double primary tumors refer to the simultaneous or sequential occurrence of 2 histologically distinct malignant tumors in the same patient.^[14–16]^ In this case, the patient had a mass in the gallbladder and a lesion in the CBD. The following aspects helped us differentiate it from double primary tumors. First of all, preoperative CT and magnetic resonance cholangiopancreatography indicated a mass in the gallbladder and dilatation of the CBD and intrahepatic bile ducts, but no separate mass was found in the CBD. This suggested that the CBD lesion might be caused by GBC invading the CBD rather than a double primary tumor. Moreover, during surgery, the CBD was found to be dilated, but no separate mass or lesion was palpated in the CBD wall, which also supported the idea that it was GBC invading the CBD. The last and most important point was that postoperative pathologic results showed that both the gallbladder and CBD lesions were moderately differentiated adenocarcinomas. Importantly, the tumor in the CBD was continuous with the GBC, and there was no evidence of another independent tumor focus. This definitely ruled out the possibility of double primary tumors.

The Whipple procedure is routinely employed for carcinoma of the pancreatic head or distal CBD, but is not routinely employed for GBC.^[17]^ In this case, however, intraoperative choledochoscopy revealed a mass at the distal CBD. Consequently, the planned radical cholecystectomy was subsequently extended to include a radical pancreaticoduodenectomy, significantly exceeding the standard scope of GBC resection. This expansion of the surgical field was primarily driven by the concern for multifocal disease necessitating wider resection margins. Nevertheless, postoperative pathological analysis demonstrated that the CBD lesion did not invade the adjacent pancreas or duodenum. This finding underscores the critical need to carefully weigh the risks of potential overtreatment when making intraoperative decisions to extend the scope of resection beyond established standards. While personalized, extended radical resection may be considered for selected patients with locally advanced disease where complete resection is achievable, such decisions require judicious evaluation of the anticipated benefit against the substantial morbidity associated with extensive procedures like the Whipple. During the surgical procedure, utilizing robotic assistance allowed for precise dissection and resection of the involved structures.^[18]^ The meticulous nature of robotic surgery is particularly beneficial in complex cases such as this, where anatomical relationships are altered due to tumor invasion.^[19]^ Effective techniques employed during the operation, such as careful identification and handling of the bile duct and vascular structures, are crucial in minimizing complications like bile leaks and hemorrhage postoperatively.^[20]^ The pathological findings of moderately differentiated adenocarcinoma in this case, alongside the absence of lymph node metastasis, suggest an early-stage tumor that may respond favorably to aggressive surgical intervention. The accurate staging of the cancer is essential for determining prognosis and subsequent treatment strategies. The pathological assessment also revealed that the cancer had not invaded major vascular structures, which is a positive prognostic factor.

As minimally invasive surgery has advanced, laparoscopic techniques have been applied to selected surgical cases; nonetheless, their widespread adoption remains constrained by limited instrument articulation, poor ergonomics, two-dimensional visualization, and technical difficulties in complex dissection and reconstruction.^[21,22]^ The advent of robotic surgical platforms has offered a possible answer to several of these challenges.^[23]^ Robotic-assisted surgery provides multiple benefits, such as three-dimensional high-resolution visualization, tremor reduction, wristed instrumentation, improved surgeon comfort, and increased accuracy in hepatobiliary procedures.^[24,25]^ These benefits are especially valuable in the field of hepatobiliary and pancreatic surgery, where meticulous dissection near major vessels and precise bilioenteric reconstruction are necessary.^[26]^ Since the initial reports of robot-assisted hepatobiliary surgery, accumulating data indicate that robotic techniques may decrease intraoperative blood loss, shorten hospitalization duration, and enhance recovery while achieving similar oncologic outcomes in appropriate patients.^[27]^ Moreover, progress in fluorescence imaging, artificial intelligence, navigation tools, and digital surgical technologies has accelerated the shift of robotic hepatobiliary surgery from technical possibility to intelligent, image-guided interventions.^[28]^ A recent study documents the successful management of a complex case involving a 69-year-old male patient with hilar cholangiocarcinoma that had extended into the mid and distal portions of the CBD. The procedure performed was a robotic-assisted left hepatectomy combined with a caudate lobectomy and HPD.^[8]^ In contrast, we utilized the da Vinci Xi system to perform radical cholecystectomy and pancreaticoduodenectomy for a patient with GBC involving the CBD. The patient had GBC with direct invasion of the CBD, requiring a rare combined procedure – radical cholecystectomy plus Whipple. This “double resection” involves intricate dissection around major vessels and 2 anatomically distinct regions, posing significant technical challenges. The da Vinci Xi system was specifically chosen to address this complexity. Its multi-quadrant capability allows seamless transition between the gallbladder and pancreatic head fields without repositioning. The instruments enable precise suturing for biliary-enteric reconstruction, while 3D high-definition visualizationand tremor filtration improve accuracy in tight surgical spaces – critical for achieving safe margins and reducing complications. Unlike laparoscopic approaches (with poor ergonomics, 2D vision, and limited articulation) or open surgery (higher morbidity), the robotic platform offers enhanced precision, faster recovery, comparable oncologic outcomes, making it a powerful tool for this ultra‑complex scenario. Both cases demonstrated the feasibility and safety of the da Vinci Xi system in hepatobiliary and pancreatic surgery.

In conclusion, this case emphasizes the importance of thorough imaging studies, the advantages of robotic-assisted surgical techniques, and the need for diligent postoperative management. During the one-year postoperative follow-up period, no tumor recurrence or progression was observed. These insights contribute to the evolving understanding of GBC and underscore the necessity for continuous research and refinement in clinical practice.

## Acknowledgments

We wish to extend our sincere gratitude to the patient involved in this study for her invaluable cooperation. Furthermore, we are deeply appreciative of the support provided by the technical support team within the Department of Pathology at the Affiliated Tumor Hospital of Xinjiang Medical University.

## Author contributions

**Conceptualization:** Boqing Wang, Guodong Chen, Kai Xu.

**Data curation:** Guodong Chen, Deliang Cao.

**Formal analysis:** Kai Xu, Chen Liu, Liping Liang, Deliang Cao.

**Funding acquisition:** Boqing Wang, Guodong Chen.

**Investigation:** Chen Liu, Jintian Tang.

**Methodology:** Wanghong Li, Chao Yi.

**Project administration:** Guodong Chen, Boqing Wang.

**Resources:** Guilin Cao, Longhua Zhang, Chao Yi, Feng Xue, Boqing Wang.

**Software:** Chen Liu, Xin Li.

**Supervision:** Wanghong Li, Jintian Tang.

**Validation:** Guilin Cao, Longhua Zhang, Jintian Tang.

**Visualization:** Guodong Chen, Xin Li, Chao Yi, Feng Xue, Boqing Wang.

**Writing – original draft:** Kai Xu, Boqing Wang.

**Writing – review & editing:** Kai Xu, Boqing Wang, Guodong Chen.
